# Anti-SRP immune-mediated necrotizing myopathy: A critical review of current concepts

**DOI:** 10.3389/fimmu.2022.1019972

**Published:** 2022-10-13

**Authors:** Xue Ma, Bi-Tao Bu

**Affiliations:** ^1^ Department of Neurology, Tangdu Hospital, Air Force Medical University, Xi’an, China; ^2^ Department of Neurology, Tongji Hospital, Tongji Medical College, Huazhong University of Science and Technology, Wuhan, China

**Keywords:** anti-SRP autoantibodies, immune-mediated necrotizing myopathy, cardiac involvement, ER stress, refractory

## Abstract

**Purpose of review:**

This review aims to describe clinical and histological features, treatment, and prognosis in patients with anti-signal recognition particle (SRP) autoantibodies positive immune-mediated necrotizing myopathy (SRP-IMNM) based on previous findings.

**Previous findings:**

Anti-SRP autoantibodies are specific in IMNM. Humoral autoimmune and inflammatory responses are the main autoimmune characteristics of SRP-IMNM. SRP-IMNM is clinically characterized by acute or subacute, moderately severe, symmetrical proximal weakness. Younger patients with SRP-IMNM tend to have more severe clinical symptoms. Patients with SRP-IMNM may be vulnerable to cardiac involvement, which ought to be regularly monitored and cardiac magnetic resonance imaging is the recommended detection method. The pathological features of SRP-IMNM are patchy or diffuse myonecrosis and myoregeneration accompanied by a paucity of inflammatory infiltrates. Endoplasmic reticulum stress-induced autophagy pathway and necroptosis are activated in skeletal muscle of SRP-IMNM. Treatment of refractory SRP-IMNM encounters resistance and warrants further investigation.

**Summary:**

Anti-SRP autoantibodies define a unique population of IMNM patients. The immune and non-immune pathophysiological mechanisms are involved in SRP-IMNM.

## Introduction

As early as 1975, Bohan and Peter divided inflammatory myopathies into two subtypes, dermatomyositis (DM) and polymyositis (PM) according to the presence of typical skin lesions and common histopathology (1). At present, based on clinical manifestations, serum myositis-specific autoantibodies, and pathological characteristics, idiopathic inflammatory myopathies (IIM) are mainly classified into 4 subgroups: DM, immune-mediated necrotizing myopathy (IMNM), anti-synthetase syndrome, and sporadic inclusion body myositis (sIBM) (2, 3). IMNM has been recently described as a distinct form of IIM. IMNM is characterized by proximal weakness, prominent or scatter myonecrosis, and a paucity of or few lymphocyte infiltrates in muscle biopsy (4).

Currently, two principle categories of IMNM, anti-signal recognition particle (SRP) autoantibodies-IMNM (SRP-IMNM) and anti-3-hydroxy-3-methylglutaryl-coa reductase (HMGCR) autoantibodies-IMNM (HMGCR-IMNM) (5, 6), account for the largest proportion of IMNM and are relatively the most described. Other subtypes, including seronegative IMNM, connective tissue disease-related IMNM, statin-related IMNM, cancer-related IMNM, and immune checkpoint inhibitors-induced IMNM are also reported (7–12). The detection of serum anti-SRP autoantibodies in IIM is much earlier than that of anti-HMGCR autoantibodies. Serum anti-SRP autoantibodies in IIM were first detected in 1982 (13, 14) and anti-HMGCR autoantibodies were first detected in 2010-2011 (15, 16). Here, we review manifestations of SRP-IMNM and highlight recent clinical and pathological advances in SRP-IMNM.

## Anti-SRP autoantibodies

SRP, a cytosolic evolutionarily conserved ribonucleotide protein, is located in the endoplasmic reticulum (ER) and consists of six distinct polypeptides, including 9, 14, 19, 54, 68, and 72KDa, binding to a 7S RNA molecule (17, 18). The SRP guides newly synthesized polypeptides to the ER for posttranslational modifications (18). Although it is believed that anti-SRP autoantibodies are associated with polymyositis in previous studies (13, 19), anti-SRP autoantibodies are specific to a unique entity of IIM featuring myofiber necrosis, myofiber regeneration, and minimal inflammation, which is now known as IMNM (20, 21). However, mechanisms of serum anti-SRP autoantibodies production in IMNM remain unclear. HMGCR is also located in ER and is responsible for cholesterol biosynthesis (22, 23). Statins may trigger autoimmune dysregulation to induce the formation of anti-HMGCR autoantibodies in a small percentage of HMGCR-IMNM. The titer of anti-HMGCR autoantibodies is significantly associated with serum creatine kinase (CK) and clinical severity in HMGCR-myopathy with statins exposure but not in patients without a history of statins (24). Statins are likely to have no association with the development of SRP-IMNM. The relationship between serum CK levels and anti-SRP autoantibodies in SRP-IMNM has not yet been elucidated. One study indicates no significant correlations between CK levels and the titer of anti-SRP54 autoantibodies (25). However, another study shows that autoantibody levels are dramatically associated with CK levels in patients with SRP-IMNM receiving therapy (26).

*In vitro* and *in vivo* experiments reveal that anti-SRP autoantibodies isolated from patients with SRP-IMNM are potentially pathogenic and target skeletal muscle fibers. Anti-SRP autoantibodies can impair myoblast regeneration, result in myofiber atrophy, and increase the production of reactive oxygen species (27). In anti-SRP autoantibodies adoptively transferred mice, skeletal muscle fiber necrosis can be detected and may be mediated by the mechanism of complement-dependent cytotoxicity (28). It remains to be further confirmed whether SRP antigen targets can be ectopically expressed on myofiber sarcolemma and under what conditions this phenomenon occurs.

## Epidemiology

IIM is a rare disease, with an incidence rate of 9-14/100000 in European countries (29, 30). IMNM comprises 20-38% of IIM (31). SRP-IMNM accounts for 18-39% of IMNM (32). The mean age of SRP-IMNM onset is 40-50 years old and it affects more females than males with a ratio of 1.6-3.6 (25, 33–35). SRP-IMNM is seldom reported in children or juveniles (36–38). Differentiating SRP-IMNM from muscular dystrophy is of critical clinical importance, as SRP-IMNM and limb-girdle muscular dystrophy (LGMD) exhibit similar clinicopathological presentations at times (38–40). These patients can be identified as serum anti-SRP autoantibodies positivity and have a favorable response to immunotherapy. HMGCR-IMNM patients with a family history of cardiomyopathy or myopathy occasionally present a chronic progressive course of weakness, which resembles other acquired myopathy or inherited myopathy and these patients may be misdiagnosed as LGMD (41).

## Clinical symptoms: Focus on the difference between SRP-IMNM and HMGCR-IMNM

### Muscular phenotype

Patients with SRP-IMNM commonly struggle to lift arms and/or squat to stand up, and occasionally find it hard to raise their head (32). SRP-IMNM is clinically characterized by acute or subacute, moderately severe, symmetrical proximal weakness, partially accompanied by myalgia, dyspnea, dysphagia, and muscle atrophy (6, 25, 34, 35, 40, 42). Distal leg, bulbar and axial muscles are incidentally involved in SRP-IMNM (25, 43).Compared to SRP-autoantibodies-negative patients, patients with SRP-IMNM tend to have facial weakness and age at onset is lower (32). The younger patients at onset seem to have more severe clinical symptoms in SRP-IMNM (42, 44). In European countries and Japan, compared to HMGCR-IMNM, the muscle weakness appears to be more severe in SRP-IMNM (6, 42). Neck weakness, dysphagia, respiratory insufficiency, and muscle atrophy occur more frequently in SRP-IMNM than in HMGCR-IMNM (6). Seronegative IMNM patients are more likely to suffer from myalgia in the Chinese population compared to SRP-IMNM and HMGCR-IMNM (7).

A highly elevated CK level is prominent in SRP-IMNM, usually more than 1000 IU/L (7, 25, 34, 40, 42). Serum CK levels positively correlate with myofiber necrosis (45). A patient being asymptomatic accompanied by an elevated serum CK is rarely observed in SRP-IMNM (46), but is as well reported in HMGCR-IMNM (47).

Human leukocyte antigens DRB1*08:03, B*5001, and DQA1*0104 are more frequently detected in SRP-IMNM and DRB1*11:01 is more prevalent in HMGCR-IMNM (48–51). These antigens derived by DRB1 alleles polymorphism may play key roles in the autoimmunity in IMNM.

### Extramuscular phenotype

There are some conflicting conclusions on cardiac involvement in SRP-IMNM. One case with SRP-IMNM was complicated by cardiomyopathy, gradually developed heart failure, and is ultimately relieved after heart transplantation (52). Some case reports and studies suggest that patients with SRP-IMNM are susceptible to subclinical myocardial damage (32, 52–55). Echocardiogram abnormalities usually appear in SRP-IMNM and account for 61% (25 of 41 patients), most presenting as diastolic dysfunction (32). On the other hand, a low risk of cardiac involvement in SRP-IMNM (2 of 16 patients, 13%) is found (21). Myocardial involvement is an important prognostic indicator for patients. Therefore, further prospective multi-center large-sample studies are required to confirm the degree of vulnerability to cardiac abnormalities in SRP-IMNM. Cardiac magnetic resonance imaging (MRI), as a highly sensitive method, is commonly recommended for screening for myocardial damage in myositis (56–58).

Other extramuscular phenotypes are sometimes present in SRP-IMNM. A low proportion of SRP-IMNM is associated with chest pain (8%) (34), arthritis (0-17%) (21, 34, 35, 43), arthralgia (39%) (34), Sicca syndrome (8%) (34), mechanic’s hand (14%) (43), and carpal tunnel syndrome (10-20%) (34, 43). Approximately 20% of patients with SRP-IMNM have ILD (6, 21, 42). Patients with SRP-IMNM seldomly display skin rash (3-6%) (6, 25, 35, 42). A cutaneous lesion occurs more frequently in HMGCR-IMNM (59). Cancer-associated SRP-IMNM is occasionally reported (6, 7, 25). Nevertheless, compared with HMGCR-IMNM and MSA-negative-IMNM, SRP-IMNM have a lower risk of tumor (10, 60). Cancer association is considered a risk factor for the development of HMGCR-IMNM (60). These extramuscular presentations may be key factors affecting the prognosis of SRP-IMNM, especially cancer and ILD.

### Muscle MRI

**Figure 1:**
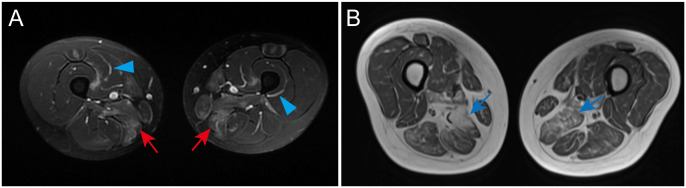
Examples of a thigh and lower leg MRI in SRP-IMNM. **(A)**, a T2-weighted image shows obvious edema in the adductor magnus, gracilis, and sartorius of the thigh (red arrow) and fascial edema (blue arrowhead). **(B)**, a T1-weighted image reveals evident muscle atrophy and fatty replacement in the posterior muscles of the thigh (blue arrow). These pictures are cited from our previous study (7).

SRP-IMNM features focal or diffuse muscle edema, atrophy, and fatty infiltration predominantly on a proximal lower extremities muscle MRI scan (61, 62) (**Figures 1A, B**). Compared to DM, fascial edema is less frequently observed in SRP-IMNM (**Figure 1A**) (61). Distal lower extremities on T1-weighted or T2-weighted images are less studied. Compared to DM, PM, and HMGCR-IMNM, muscle abnormalities are more diffuse and common in SRP-IMNM on a thigh muscle MRI scan (61). Of note, the degree of muscle edema is not significantly associated with the disease severity (61). In addition, rapid fat infiltration on an MRI scan may be a risk factor associated with refractory SRP-IMNM (44).

## Histopathological manifestations

### Typical myopathological findings

**Figure 2:**
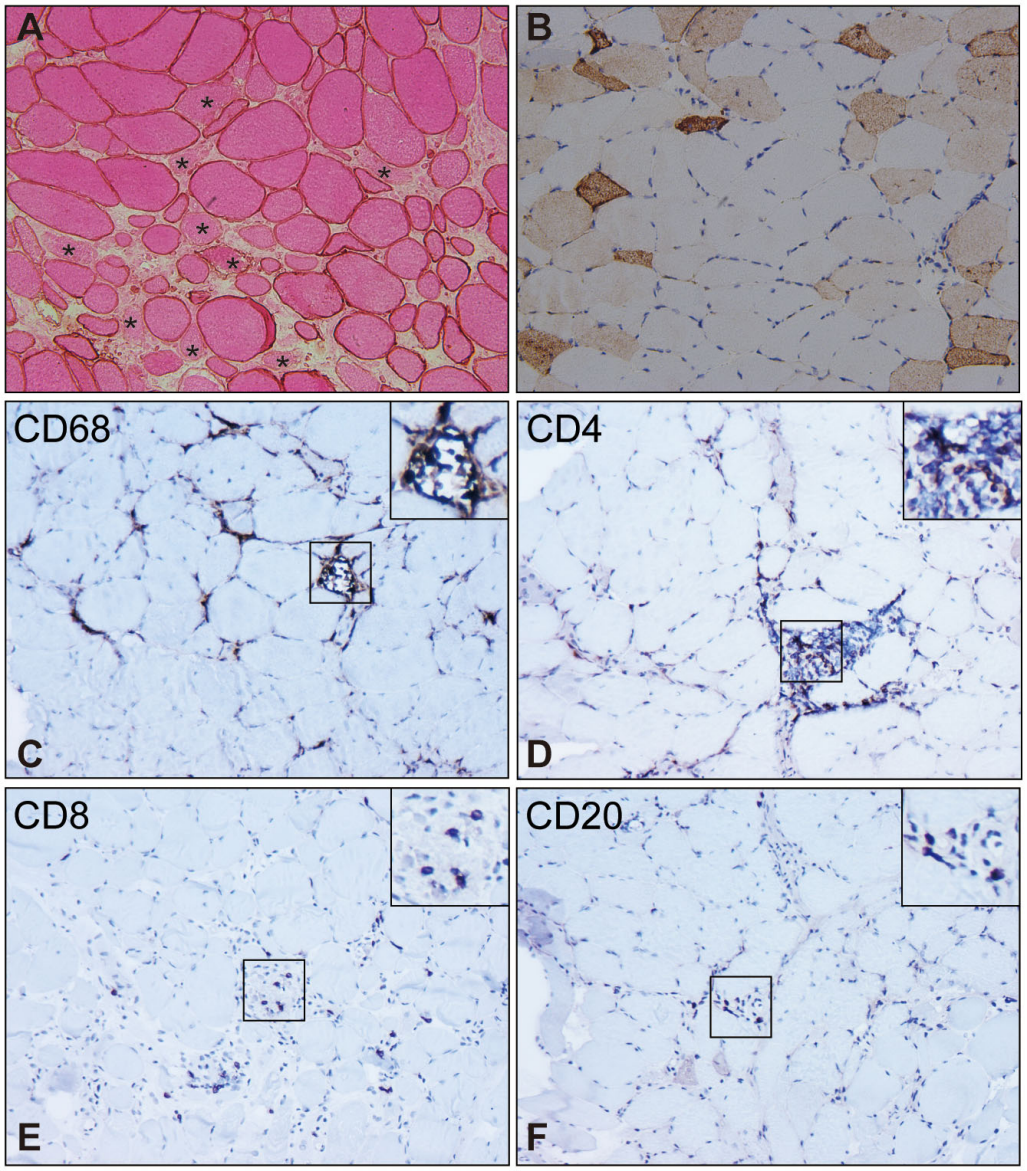
Pathological characteristics of muscle biopsy in SRP-IMNM. **(A)**, dystrophin combined with eosin staining reveals necrosis myofibers (asterisk). **(B)**, immunohistochemical staining of Neural Cell Adhesion Molecule1 (NCAM1)/CD56 shows scattered myofiber regeneration. Inflammatory cell analysis demonstrates scattered or focal CD68^+^ macrophage infiltration and myophagocytosis **(C)**, scattered CD4^+^ **(D)** and CD8^+^ T lymphocyte **(E)**, and a few CD20^+^B lymphocytes **(F)**. Magnification **(A-F)**: 200x. These pictures are cited from our previous study (7, 63).

SRP-IMNM is pathologically characterized by patchy or diffuse myonecrosis and myoregeneration (**Figures 2A, B**), accompanied by a paucity of inflammatory infiltrates (**Figures 2C–F**) (40, 64, 65). Myofiber regeneration is considered the physiologic consequence of necrosis and is essential for muscle restoration in skeletal muscle disease (66). The percentage of necrotic myofibers significantly correlates with the percentage of regenerating myofibers in SRP-IMNM (45).

To date, the pathological mechanisms of SRP-IMNM are mainly focused on the inflammatory and autoimmunity response. Type 1 helper T cell/classically activated macrophage M1 response derived inflammation is a predominant pathological finding of SRP-IMNM (67). A larger number of endomysial lymphocytic infiltration is rare in specimens from SRP-IMNM (45). SRP-IMNM exhibits diffuse major histocompatibility complex class I (MHC-I) positivity on sarcolemma and scatter membrane attack complex (MAC) deposition on non-necrotic myofibers in muscle specimens (45, 65, 67) A previous study shows that upregulation of MHC-I on the surface of muscle cells induces clinical, histological, and immunological manifestations similar to human myositis in young mice (68). There is a positive correlation between the percentage of MAC deposited fibers and the percentage of necrotic myofibers, suggesting a complement-mediated mechanism in SRP-IMNM (45). Furthermore, dysregulated T cells and the programmed death-1 pathway are described in SRP-IMNM (69). High mobility group box protein 1 (HMGB1), a ubiquitous non-histone nuclear DNA-binding molecule, might play a pro-inflammatory role under disease conditions. The highly sarcoplasmic HMGB1 expression is positively associated with myofiber autophagy, muscle inflammation, myonecrosis, myoregeneration, and muscle weakness in SRP-IMNM (70). Decreased vascular density and enlargement of endomysial capillaries are previously reported in muscle from SRP-IMNM, which may be relevant to ischemia-induced damages (20). These data indicate that autoimmune and inflammatory responses contribute to the pathogenesis of SRP-IMNM to a great extent.

Non-immune mechanisms have been explored in IMNM. A previous study indicates that the upregulation of MHC-I expression on myofibers elicits the elevation of ER stress marker, a glucose-regulated protein 78 (GRP78)/immunoglobulin heavy chain binding protein (BiP) in mice (71), suggesting a close relationship between ER stress and the up-regulation of MHC-I in IMNM. Intriguingly, a scattered GRP78/BiP sarcoplasmic expression is detected in muscle specimens from IMNM. Moreover, BiP expression significantly correlates with myofiber autophagy, myonecrosis, myoregeneration, and clinical disease severity in IMNM (63). Another study also implies ER stress is a key pathological mechanism in IMNM (72). The autophagy marker SQSTM1/p62 immunopositivity with large, rimmed vacuoles is considered a pathological feature in sIBM (73), which is different from SQSTM1/p62 fine granular and homogeneous staining in the sarcoplasm of IMNM (74, 75). Other autolysosome markers, including LC3 and LAMP2, also demonstrate a diffuse sarcoplasmic staining pattern in SRP-IMNM (75). Acid phosphatase staining shows randomly distributed lysosomal activation in scattered myofibers (6). Mitophagy may play a role in HMGCR-IMNM (76) and has not been studied in SRP-IMNM. In addition, necroptosis may be involved in myofiber death in SRP-IMNM (77).

## Atypical pathological findings in muscle

Atypical pathological manifestations, including significant mitochondrial abnormality, myofibrillary pathological changes, and granulomatous inflammation, occasionally occur in SRP-IMNM (41). HMGCR-IMNM patients with a disease duration of over three years may resemble LGMD on skeletal muscle pathological presentations (78). Recent studies show unusual pathological changes in damaged HMGCR-IMNM, including perimysium and myofibrillary changes (79) and an increased presence of apoptosis marker B-cell lymphoma 2-positive T-lymphocytes (80), which so far have not been recognized in SRP-IMNM.

## Electromyography

Electromyography is an effective examination for distinguishing myogenic damage from a neuromuscular junction and neurogenic damage. SRP-IMNM is characterized by typical myogenic damage, presenting as a positive spike of fibrillation potential in proximal limbs, early recruitment of motor unit potential (20, 32), and prominent spontaneous potential (35). A myotonic potential is sometimes observed in SRP-IMNM (32).

## Treatment and prognosis

### General treatment for SRP-IMNM

Currently, clinical randomized trials and large sample-sized literature are lacking, making it difficult to reach definite conclusions on the treatment strategies of IMNM. According to the recommendations from the 224th European Neuromuscular Centre (ENMC) International Workshop, initial treatment for SRP-IMNM usually starts with intravenous and/or oral glucocorticoids (4). Depending on the disease severity and response to glucocorticoids monotherapy, the treatment can be supplemented with other immunotherapy at the same time or within one month, such as immunosuppressants, intravenous immunoglobulin (IVIG), and/or rituximab (4). The goal of maintenance treatment is to minimize the symptoms with the lowest dose of glucocorticoids. Generally, steroid monotherapy does not control the disease progression and most patients required additional immunosuppressants to achieve improvement in IMNM (4, 6, 7, 81). A high recurrence risk by decreasing the dose of glucocorticoids is reported (25, 32, 52). Once dyspnea occurs in IMNM, intensive care and augmenting immunotherapy are required, including plasma exchange, cyclophosphamide, and/or cyclosporine (4). Anti-B cell therapies, belimumab (82) and rituximab, seem to be relatively safe and effective medications for the majority of patients with SRP-IMNM (42, 83, 84). The early use of IVIG (85) or co-administration of tacrolimus with corticosteroids (86) dramatically decreases the dose of steroids and improves the symptoms of patients with SRP-IMNM.

There are discrepancies among several studies on the prognosis of patients with IMNM. In 224th ENMC International Workshop, SRP-IMNM is regarded as one of the most disabling IIMs, and patients often have poor muscle recovery even with treatment (4). A prior study indicates that 50% of patients with SRP-IMNM achieve satisfactory outcomes with immunotherapy after 4 years, and most patients’ serum CK levels are not restored to normal (42). On the other hand, some studies reveal that most patients with SRP-IMNM obtain satisfactory improvement on formal immunotherapy (7, 21, 86).

### Treatment for refractory IMNM

Data are limited concerning the treatment of refractory IMNM. The definition of refractory IMNM is not explicit. When the treatment with glucocorticoids with immunosuppressants at a known effective dose is performed for at least three to twelve months, and muscle weakness is still worsening or not better, these patients can be deemed to be refractory (44, 87). There are some risk factors associated with refractory SRP-IMNM, including being male, severe muscle weakness, concurrent ILD, quick development of muscle fatty infiltration, and more B cell activating factor receptor and B lymphocyte infiltration in muscle specimens (44). Rituximab may be an effective treatment strategy against refractory IMNM (83, 84). Some patients with refractory IMNM respond well to tocilizumab (87). High-dose cyclophosphamide is effective for several refractory IMNM patients (88). In addition, a refractory SRP-IMNM patient responds well to myeloid autologous stem cell transplantation (89).

## Conclusion

SRP-IMNM is clinically characterized by acute or subacute proximal extremities weakness at the onset. Autoimmune and inflammatory responses play key roles in the pathological mechanism. In addition, ER stress-induced autophagy pathway and necroptosis are involved in the muscular pathogenesis of SRP-IMNM. Most patients with SRP-IMNM at the acute or subacute stage respond well to high-dose steroid therapy. Steroids combined with immunosuppressive agents are recommended to be applied during maintenance therapy. It is required to regularly monitor the disease progression, especially extramuscular manifestations, including cardiac involvement and ILD. The treatment of refractory SRP-IMNM still needs further exploration.

## Author contributions

XM contributed to the body, provided the figures, designed the write-up, and made the required changes. B-TB did the critical review and editing. XM and B-TB approved the final manuscript.

## Funding

This study was supported by the National Natural Science Foundation of China (Grant Number: 81873758).

## Conflict of interest

The authors declare that the research was conducted in the absence of any commercial or financial relationships that could be construed as a potential conflict of interest.

## Publisher’s note

All claims expressed in this article are solely those of the authors and do not necessarily represent those of their affiliated organizations, or those of the publisher, the editors and the reviewers. Any product that may be evaluated in this article, or claim that may be made by its manufacturer, is not guaranteed or endorsed by the publisher.
